# EFFECT OF HEARING INTERVENTION ON OLDER ADULTS AT RISK OF COGNITIVE DECLINE: ACHIEVE RANDOMIZED TRIAL

**DOI:** 10.1093/geroni/igae098.0057

**Published:** 2024-12-31

**Authors:** James Pike

**Affiliations:** New York University, New York, New York, United States

## Abstract

The Aging and Cognitive Health Evaluation in Elders (ACHIEVE, Clinicaltrials.gov Identifier: NCT03243422) randomized trial investigated the effect of a hearing intervention versus a health education control on 3-year cognitive change among dementia-free older adults with untreated hearing loss. Participants were recruited from the Atherosclerosis Risk in Communities (ARIC) study (n=238) or de novo from the community (n=739). The hearing intervention slowed cognitive decline by 48% in ARIC participants, but not in healthy de novo volunteers. A possible explanation for this difference is that the cognitive benefits of the hearing intervention could be observed only for individuals at increased risk for cognitive decline. To evaluate whether risk of cognitive decline moderated the effect of the hearing intervention, we used a sample of dementia-free ARIC participants (N=2,692) who did not participate in ACHIEVE to develop a model that explained 80.7% of the variance in cognitive decline over a 6-year period. The model was applied to baseline measures of ACHIEVE participants (N=977) to calculate their predicted risk of cognitive decline. The covariate-adjusted, intention-to-treat effect of the hearing intervention on 3-year cognitive change was examined in a mixed effects model that included a three-way interaction between time, randomization, and predicted risk of cognitive decline. Among participants in the top quartile of risk, cognitive decline in the hearing intervention group was 58.1% (95% CI 31.4%-90.9%) slower than the control group. The results suggest the effect of a hearing intervention on 3-year cognitive change was greatest among individuals at higher risk of cognitive decline.

